# Inherited risk plus prenatal insult caused malignant dysfunction in mesenteric arteries in adolescent SHR offspring

**DOI:** 10.1371/journal.pone.0215994

**Published:** 2019-04-24

**Authors:** Yuan Zhong, Xueqin Feng, Ting Xu, Chunli Yang, Wenna Zhang, Xueyi Chen, Xiaorong Fan, Likui Lu, Meng Zhang, Lingjun Li, Zhice Xu

**Affiliations:** 1 Institute for Fetology, First Hospital of Soochow University, Suzhou, Jiangsu, China; 2 Obstetrics and Gynecology, Municipal Hospital, Suzhou, Jiangsu, China; 3 Obstetrics and Gynecology, Tengzhou Central People’s Hospital, Zaozhuang, Shandong, China; 4 Center for Prenatal Biology, Loma Linda University, Loma Linda, CA, United States of America; Max Delbruck Centrum fur Molekulare Medizin Berlin Buch, GERMANY

## Abstract

Prenatal hypoxia can induce cardiovascular diseases in the offspring. This study determined whether and how prenatal hypoxia may cause malignant hypertension and impaired vascular functions in spontaneous hypertension rat (SHR) offspring at adolescent stage. Pregnant SHR were placed in a hypoxic chamber (11% O_2_) or normal environment (21% O_2_) from gestational day 6 until birth. Body weight and blood pressure (BP) of SHR offspring were measured every week from 5 weeks old. Mesenteric arteries were tested. Gestational hypoxia resulted in growth restriction during 6–12 weeks and a significant elevation in systolic pressure in adolescent offspring at 12 weeks old. Notably, endothelial vasodilatation of mesenteric arteries was impaired in SHR adolescent offspring exposed to prenatal hypoxia, vascular responses to acetylcholine (ACh) and sodium nitroprusside (SNP) were reduced, as well as plasma nitric oxide levels and expression of endothelial nitric oxide synthase (eNOS) in vessels were decreased. Moreover, mesenteric arteries in SHR offspring following prenatal hypoxia showed enhanced constriction responses to phenylephrine (PE), associated with up-regulated activities of L-type calcium channel (Ca^2+^-dependent), RhoA/Rock pathway signaling (Ca^2+^-sensitization), and intracellular Ca^2+^ flow. Pressurized myograph demonstrated altered mechanical properties with aggravated stiffness in vessels, while histological analysis revealed vascular structural disorganization in prenatal hypoxia offspring. The results demonstrated that blood pressure and vascular function in young SHR offspring were affected by prenatal hypoxia, providing new information on development of hypertension in adolescent offspring with inherited hypertensive backgrounds.

## Introduction

Hypertension, affecting more than 25% of adult ≥30 years old globally [1], is also a great problem in children and adolescents [2–4]. It has been widely accepted that interaction of genetic and environmental factors is critical for development of hypertension [3]. Previous studies using inherited hypertensive models has revealed that adverse prenatal influences, including exposure to nicotine [4], malnutrition [5,6] and high salt diets [7] negatively impacted on cardiovascular systems in young offspring. However, it is unknown whether and how hypoxia during pregnancy may affect blood pressure at adolescent stage in the offspring with hypertension-related genetical defects. Answering this question would further increase our understanding how genetic and environmental interactions impacts on development of hypertension.

Notably, *In utero* hypoxia is a well established clinical stress that can increase risks of hypertension in later life in human or experimental animals [8–10]. Hypoxia is one of common complications during pregnancy. Besides high attitudes, many clinical conditions, including preeclampsia, abnormal placenta or umbilical cord, may induce *in utero* hypoxia and fetal growth restriction [11,12]. Hypoxia during pregnancy could significantly increase risks of hypertension in adult offspring [8,9], not in younger offspring. Although prenatal hypoxia might alter vascular functions, baseline BP is usually not significantly increased in most genetically normal rodents during adolescent periods [10,13,14]. Future studies could consider using telemetry approach to measure BP [15]. It remains unknown whether prenatal hypoxia might cause hypertension in the adolescent with inherited hypertensive genes. It would be very interesting to use inherited hypertensive model to test if hypoxia during critical developmental fetal life would change appearance time of hypertension at younger ages.

Spontaneously hypertensive rats (SHR) have been one of the most appropriate inherited hypertensive models for studies of essential hypertension since 1960s. In the present study, prenatal hypoxia was an adverse environmental factor. SHR was a model with genetic defects in cardiovascular systems. We hypothesized that prenatal environmental insult plus genetic defects would make worse for vascular functions as well as BP in adolescent SHR offspring. The fundamental hemodynamic characteristic of hypertension is a remarkable increment in peripheral vascular resistance. Mesenteric arteries (MA, intra lumen 100-500um) are the most typical resistance arteries that play important roles in BP [16]. Previous experiments using adult offspring born from mothers with normal BP and hypoxia in pregnancy have demonstrated MA impairments such as augmented vasoconstrictions to vascular agonists [17], endothelial dysfunction [18], and aggravated arterial stiffness [19]. However, there has been very limited information regarding effects and mechanisms of prenatal hypoxia on vascular functions and BP in adolescent offspring with genetic defects. To gain new knowledge in that part would increase our understanding of essential hypertension in developmental origins, and probably help to find clues and targets for early prevention or treatments of the vascular disease.

## Methods

### Animals

SHR (12–16 weeks) were obtained from Vital River Laboratories, Beijing, China. Rats were allowed free access to commercial food and tap water and housed in a 12:12 light–dark cycle. After 1 week of recovery of transportation, one female rat mated with two male rats. Pregnancy was confirmed by detecting vaginal mucus plugs and the day was designated as the first day of gestation. Pregnant SHR were randomly divided into hypoxia group (11% oxygen) that was induced hypoxia by a mixture of nitrogen gas and room air in an individual chamber from gestational day 6 to 21 and the control group (21% oxygen) that was housed with room air flowing through the same chambers. All the animals were allowed to give birth naturally. Pups (n = 5–7 per dam) were kept with their mothers until weaning. After weaning, male offspring (n = 3–4 per dam) from both groups was used for further experiment. Pregnant SHR was kept alone in one cage and three male offspring were put into one cage. The litters and individual rats were randomly selected for experiments. All experimental procedures were approved by the Ethical Committee of First Hospital of Soochow University and the Institutional Animal Care Committee and in accordance with the Guide for the Care and Use of Laboratory Animals (NIH Publication No. 85–23, 1996).

### Measurements of blood pressure, body and organ weight

Ten offspring from different litters each group were measured blood pressure by tail-cuff plethysmographic method. The rats were handled and pre-trained each day for one week by the researchers so that the rats got used to hands of researchers and the experimental environments/materials, including the cuffs and measuring procedure before BP testing. Each rat was tested twice a week from 5 to 12 weeks after birth within pre-warmed thermostatic cages and the mean value of BP was taken. During a testing, the mean of 5 consecutive BP values measured was taken after SP stabilization. The procedure was exactly same between the control and experimental groups. Body weight was also recorded. After 12 weeks of age, offspring were sacrificed by cervical dislocation when exposed to 5% isoflurane until failing righting reflex. The heart, kidney, spleen, and brain were removed and weighed. Mesenteric arteries were isolated for further experiments. Blood samples were collected and frozen for measurements of nitric oxide (NO) and endothelin-1 (ET-1).

### Measurements of vessel tone

Segments of small mesenteric arteries (A3) from 10 animals each group were isolated and suspended in chambers filled with HEPES-PSS solution (mmoll^-1^; NaCl 141.85, KCL 4.7, MgSO_4_ 1.7, EDTA 0.51, CaCl_2_·2H_2_O 2.79, KH_2_PO_4_ 1.17, glucose 5.0, and HEPES 10.0; pH7.4) and gassed continuously with 95% O_2_−5% CO_2_. Wire myograph (Dual Wire Myograph System, Model 410A; DMTA/S, Aarhus, Denmark) was used to measure vascular functions as previously described [20]. Cumulative concentrations of acetylcholine (ACh, 10^−12^–10^−4^ moll^-1^) or sodium nitroprusside (SNP, 10^−9^–10^−5^ moll^-1^) was used following application of PE (10^−4^ moll^-1^) or 5-HT (10^-5^moll^-1^), respectively, to maintain steady vasoconstrictions for at least 15–20 min. Phenylephrine (PE, 10^−8^–10^-4^moll^-1^) was used as a vasoconstrictor. NG-Nitro-l-arginine (L-Name, nitricoxide-synthaseinhibitor; 100μmoll^-1^), Y27632 (antagonist for Rock; 1μmoll^-1^), or nifedipine (NIFE, antagonist for L-type calcium channels; 1μmoll^-1^) was added into the chambers for 30 minutes before application of PE. The direct vasodilatation caused by cumulative nifedipine (10^−9^–10^−5^ moll^-1^) was also recorded. Bay K8644 (agonist for voltage dependent calcium channels, 10^−9^–10^-5^moll^-1^) was used for testing dose-related contractions. Each vessel ring was used once only. Signals were recorded by Power-Lab system with Chart 5 software (AD Instruments, Castle Hill, NSW, Australia). All drugs were purchased from Sigma (St. Louis, USA).

### [Ca^2+^]_i_ imaging in mesenteric arteries

Mesenteric arteries from 5 animals each group were kept in oxygenated dissociation buffer containing (mmol/L): NaCl 135, KCl 5.6, MgCl_2_ 1.0, HEPES 10, glucose 10, Na_2_HPO_4_ 0.42, NaH_2_PO_4_ 0.44, and NaHCO_3_ 4.2 (95% O_2_, 5% CO_2_, pH 7.4 with NaOH). 1-mm vessel strips was incubated in Ca^2+^ free dissociation buffer containing 4 mg/ml papain, 2 mg/ml bovine serum albumin, and 1 mg/ml dithiothreitol for 40 min at 37°C. As previously described, Ca^2+^ indicator Fura 2-AM (Calbiochem, San Diego, CA, USA) was used for [Ca^2+^]_i_ monitoring [21]. Then VSMCs were loaded in Ca^2+^-free PSS solution with fura2-AM (2 mmol/L) for 30 minutes at room temperature. [Ca^2+^]_i_ levels (in nmol/L) were calculated qualitatively by fluorescence ratio of fura-2AM at 340 and 380 nm wavelength (Ratiof340/380). [Ca^2+^]_i_ were monitored and recorded continuously using IonOptix system (IonOptix, USA) before or after PE (10^−4^ moll^-1^) was added.

### ELISA

The plasma from 5 animals each group was used for detection of NO and ET-1. Concentrations of NO (A013-2, Jiancheng, Nanjing, China) and ET-1(H093, Jiancheng, Nanjing, China) in plasma were measured with enzyme-linked immunosorbent assay (ELISA) using commercially available kits with manufacturer’s instructions. The kits have high sensitivity and excellent specificity for detection of NO and ET-1. NO, as an active chemical, would convert to NO_2_^-^ or NO_3_^-^ in vivo. The sum of NO_2_^-^ and NO_3_^-^ in the plasma could be tested accurately by NO assay. In addition, the minimum detectable dose of rat ET-1 is typically less than 0.31 ng/L. No significant cross-reactivity or interference between rat ET-1 and analogues was observed. All data were processed in a blind manner according the instructions.

### Transmission electron microscope and hematoxylin and eosin Staining analysis

Mesenteric arteries from five 12 week-old offspring each group were isolated and fixed in 1% glutaraldehyde at 4°C for 1 hour and then washed in distilled water, postfixed for 60 min. in 1% OsO4 in water and washed again in distilled water. For contrast enhancement the vessels were block stained overnight in 1.5% aqueous uranyl acetate, dehydrated through a series of ethanol concentrations and embedded in resin LX-112 (Ladd), The resin blocks were polymerized for 48 hours at a temperature of 60°C. Ultrathin sections of 90 nm were cut on a Reichert EM UC6 with a diamond knife, collected on formvar coated grids and stained with uranyl acetate and lead citrate. Finally, the vessels were examined using transmission electron microscope (JEOL-1010, Japan) at Soochow University Core Facility as described previously [22]. In addition, mesenteric arteries were also fixed in 4% formalin solution and embedded in paraffin. Sections with a thickness of 5–6μm were sliced from the paraffin‐embedded blocks and stained with hematoxylin and eosin stain. Photos were captured by Nikon microscope attached with digital camera imaging system [23,24].

### Pressure myograph

Segments of mesenteric arteries from 10 animals each group were cannulated and mounted in a perfusion chamber (Living Systems Institute, USA) with calcium-free PSS (0 Ca^2+^; omitting calcium and adding 10mM EGTA) gassed with a mixture of 95% O_2_ and 5% CO_2_. Intraluminal pressure was raised to 120 mmHg and the artery was unbuckled by adjusting the canella. The segment was then set to a pressure of 60 mmHg and allowed to equilibrate for 60 min at 37°C. Then the pressure was reduced to 3mmHg and increased by step of 20 mmHg. Internal and external diameters (ID and ED) were recorded automatically with pressure myograph setup (IonOptix, USA) over a range of pressures (3–120 mmHg). As described [25], mechanical parameters for a given pressure were calculated as follows: WT = (ED-ID)/2, CSA = (π/4)×(ED^2^-ID^2^), wall/lumen = (ED -ID)/2ID. Incremental distensibility was calculated as (ID_i_-ID_0_)/(ID_i_ ×ΔP) ×100, where ID_i_ is the observed internal diameter for a given pressure, ID_0_ is the internal diameter at 3 mmHg and ΔP is the difference between the given pressure (1 mmHg = 133.4 N/m^2^) and 3 mmHg. Circunferential wall stress = (P×ID_i_)/2WT. Young’s elastic modulus (E = stress/strain) was used for assessing mesenteric artery stiffness and an exponential model was employed to fit the stress-strain curve.

### RNA isolation and quantitative RT-PCR

Total RNA from 10 animals each group was isolated from mesenteric arteries using Tri-Reagent (Invitrogen, CA, USA) and reverse-transcribed to cDNA (cDNA RT kit; Toyobo, Japan). Real-time PCR was performed with SYBR Green detection (SYBR Green Supermix Taq Kit, Takara, Japan) on iCycler, MyiQ two Color Real-Time PCR Detection System (Bio-Rad) according to the manufacturer's instructions. The gene specific primers (Sangon, Shanghai, China) were shown in Table 1. All primers were verified to yield a single PCR product with the correct molecular weight. The PCR conditions were 5 minutes at 95°C, 40 cycles of 15 seconds at 95°C followed by 15 seconds at 60°C and 30 seconds at 72°C. ΔΔCt method was used to comparatively quantify the amount of mRNA.

**Table 1:** List of oligonucleotide primers used in this study.

Gene	Accession	Primer sequence (5’–3’)	Product length (bp)
eNOS	NM_021838.2	Forward:AGGCAATCTTCGTTCAGCCA Reverse:GTGAAGAGTTCTGGGGGCTC	249
Rock1	NM_031098.1	Forward:AATCTTCCAGTTGGTTCTGCCT Reverse:CATAGATGGACTGGATTGTTCCTT	201
Rock2	NM_013022.2	Forward:TGCCCGATCATCCCCTAGAA Reverse:GAAGGCAGTTAGCTTGGTTTGT	102
Cav1.2	NM_012517.2	Forward:TTATGGCCTTCAAACGTGGC Reverse:CGAAGGCCCGAATCATTGTG	137
β-Actin	NM_031144.3	Forward:CCCGCGAGTACAACCTTCTTG Reverse:ACAATGCCGTGTTCAATGGG	289

### Western blot

Protein abundance of Cav1.2α1c, Rock1, Rock2, RhoA and MYPT-1 in mesenteric arteries from 10 offspring each group was measured with western blot analysis normalized to ACTB. The primary antibodies were diluted (Cav1.2α1c, 1:250; Rock1,1:200; Rock2,1:500; RhoA,1:500; MYPT-1,1:250; Santa Cruz Biotech, CA, USA) and used, then rabbit anti-goat secondary antibody (1:5,000) was added for testing of Cav1.2α1c, rabbit anti-mouse secondary antibody (1:5,000) was used for testing of Rock1 and RhoA, and a goat anti-rabbit secondary antibody (1:5,000) for other proteins. Blots were developed using enhanced chemiluminescence detection reagents (Thermo, Illinois, USA), and specific bands were quantified using an imaging system (Tanon System, Shanghai, China). Imaging signals were digitized and analyzed. The ratio of band intensity to ACTB was obtained for analysis.

## Data analysis

Data are expressed as mean ± SEM. Differences were considered significant at P < 0.05 by two-tailed unpaired Student’s t-test or two-way ANOVA followed by Bonferroni test, where appropriate. Statistical analysis was carried out with GraphPad Prism 5.

## Results

### The effect of prenatal HY on weight of body and organs and blood pressure responses in the SHR offspring

**Fig 1:**
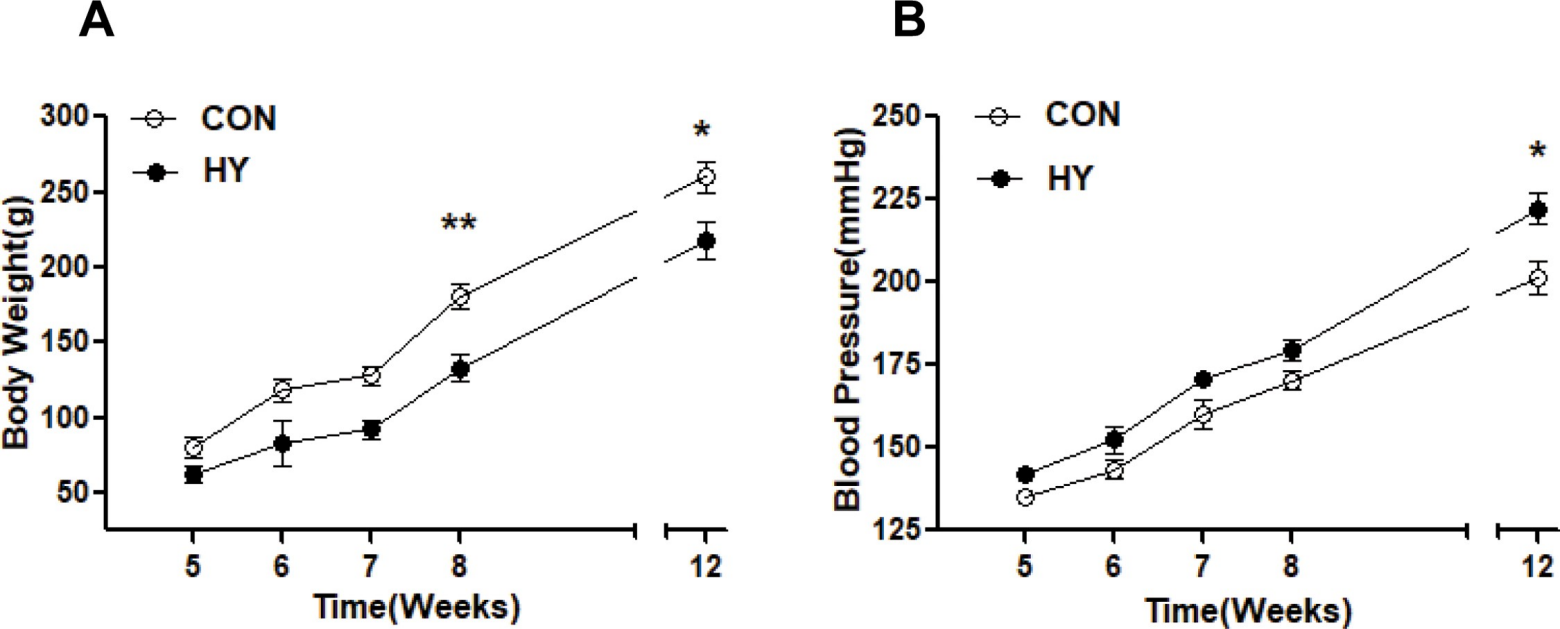
The effect of prenatal HY on vasodilatation of mesenteric arteries in adult offspring. (A) and systolic pressure (B) in SHR male offspring from 5 to 12 weeks of age. Comparisons were made using Two-way ANOVA test (n = 10 each group, *P<0.05, **P<0.01).

Hypoxia group showed a restricted body weight growth compared to CON group (Fig 1A). Significant difference was observed at 8 weeks of age (CON: 180.3±4.5g; HY: 132.6±3.9g;) and 12 weeks of age (CON: 259.6±2.6g; HY: 218.1±5.4g) (F_0.01(1,4)_ = 21.20<35.44, P<0.01<0.05). However, there was no significant difference in organ weight in 12-weeks-old offspring (Table 2).

**Table 2:** Organ weight of adolescent offspring.

	Organ weight (g)		Organ weight/Body weight	
	CON	HY	CON	HY
Heart	0.96±0.03	0.93±0.03	0.38%±0.01%	0.39%±0.01%
Brain	1.71±0.07	1.67+0.03	0.70%±0.03%	0.71%±0.02%
Kidney	0.96±0.03	0.87±0.04	0.38%±0.01%	0.37%±0.02%
Spleen	0.56±0.04	0.61±0.07	0.21%±0.01%	0.25%±0.03%

Blood pressure was measured every week from 1-month-old to 3-month old. Systolic pressure analysis showed an increased tendency in HY group compared to CON group. Notably, systolic pressure in young HY offspring was at higher levels at every measuring time each week through the monitoring period, and statistical significance was observed in 12 weeks of age (CON: 201.5±2.3mmHg; HY: 222.3±3.3mmHg; F_0.05(1,4)_ = 7.71<26.95, P<0.05, Fig 1B).

### The effect of prenatal HY on vasodilatation of mesenteric arteries in adult offspring

**Fig 2:**
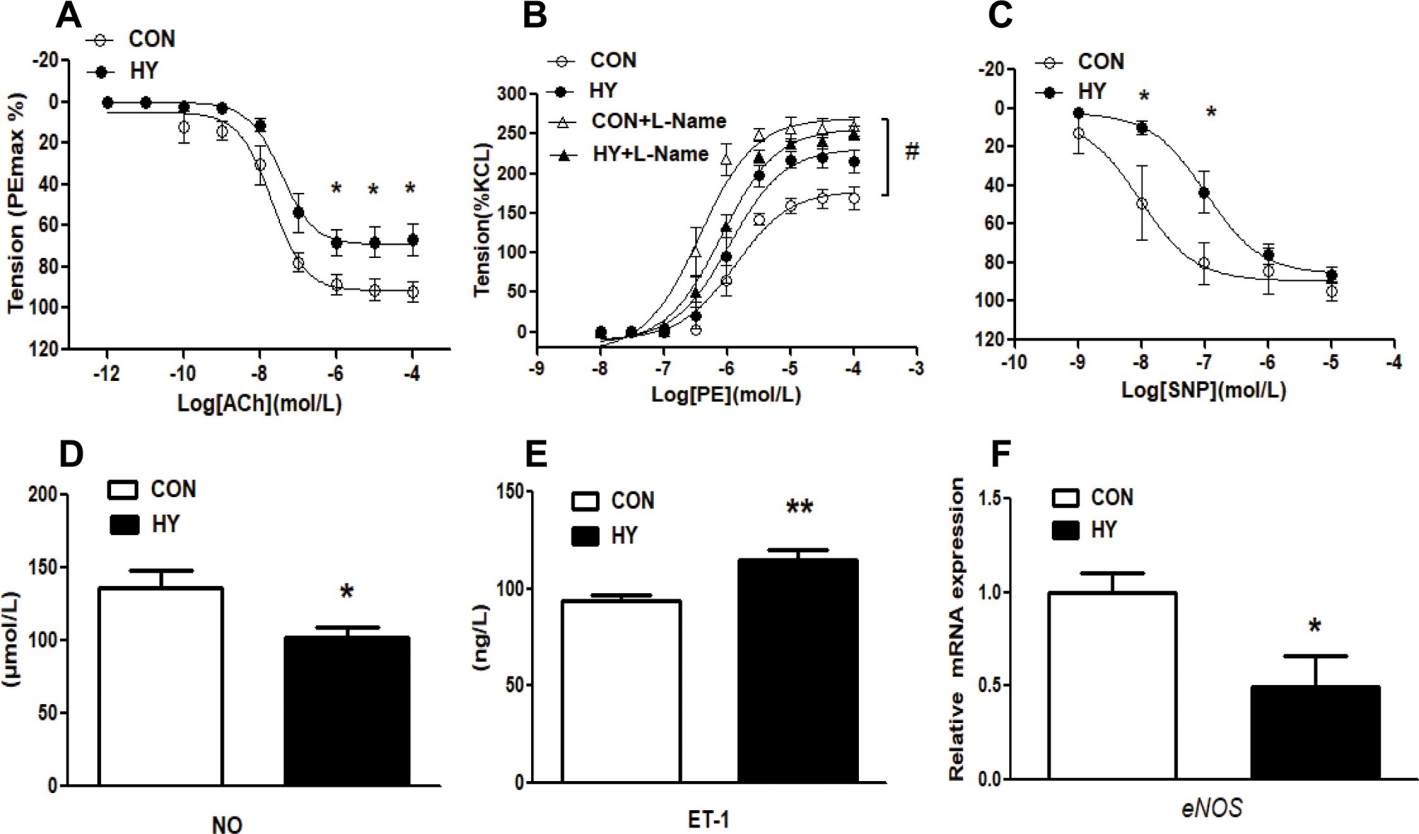
The effect of prenatal HY on vasodilatation of mesenteric arteries in adult offspring. (A) Acetylcholine (ACh)-mediated vasodilatation. The arteries were contracted with phenylephrine (PE) 10^−4^ M in prior. Two-way ANOVA test was used (n = 10, *P<0.05). (B) PE-increased vasoconstriction in presence or absence of NG-Nitro-l-arginine(L-Name). Comparisons were made using Two-way ANOVA test (n = 10, ^#^P<0.05). (C) Sodium Nitroprusside (SNP)-mediated relaxation in mesenteric arteries in 5-Hydroxytryptamine (5-HT)-induced contraction. Comparisons were made using Two-way ANOVA test (n = 10, *P<0.05). NO (D) and ET-1 (E) in plasma from 12-week-old offspring. Comparisons were made using two-tailed unpaired t-tests (n = 5, *P<0.05). (F) Relative mRNA expression of eNOS in mesenteric arteries. Comparisons were made using two-tailed unpaired t-tests (n = 10, *P<0.05).

Endothelial vasodilatation of mesenteric arteries was tested with cumulative ACh. ACh-mediated relaxation response was much weaker in HY group (F_0.05(1,8)_ = 5.32<34.10, P<0.05, Fig 2A). Nitricoxide synthase inhibitor L-Name elevated the constriction by PE in both groups, while significant difference was observed in CON group (F_0.05(3,8)_ = 4.07<46.71, P<0.05, Fig 2B). HY group showed a weaker endothelial function. Additionally, endothelium-independent relaxation was also assessed with SNP. After pre-contraction induced by 5-HT, the curve of dilatation response of SNP shifted to right in HY group to that in CON group (F_0.05(1,4)_ = 7.71<15.02, P<0.05, Fig 2C). In addition, the plasma NO was decreased in HY group (CON: 135.9 ±11.9 μmol/L; HY: 100.4 ±8.1 μmol/L), while ET-1 in plasma was increased compared with CON group (CON: 93.6±2.9 ng/L; HY: 115.2±5.1 ng/L) (P<0.05, Fig 2D and 2E). The relative mRNA expression level of *eNOS* was decreased remarkably compared with CON group (Fig 2F). It indicated that endothelium-dependent and endothelium-independent relaxation function in HY group was impaired.

### The effect of prenatal HY on contraction of mesenteric arteries in adult offspring

**Fig 3:**
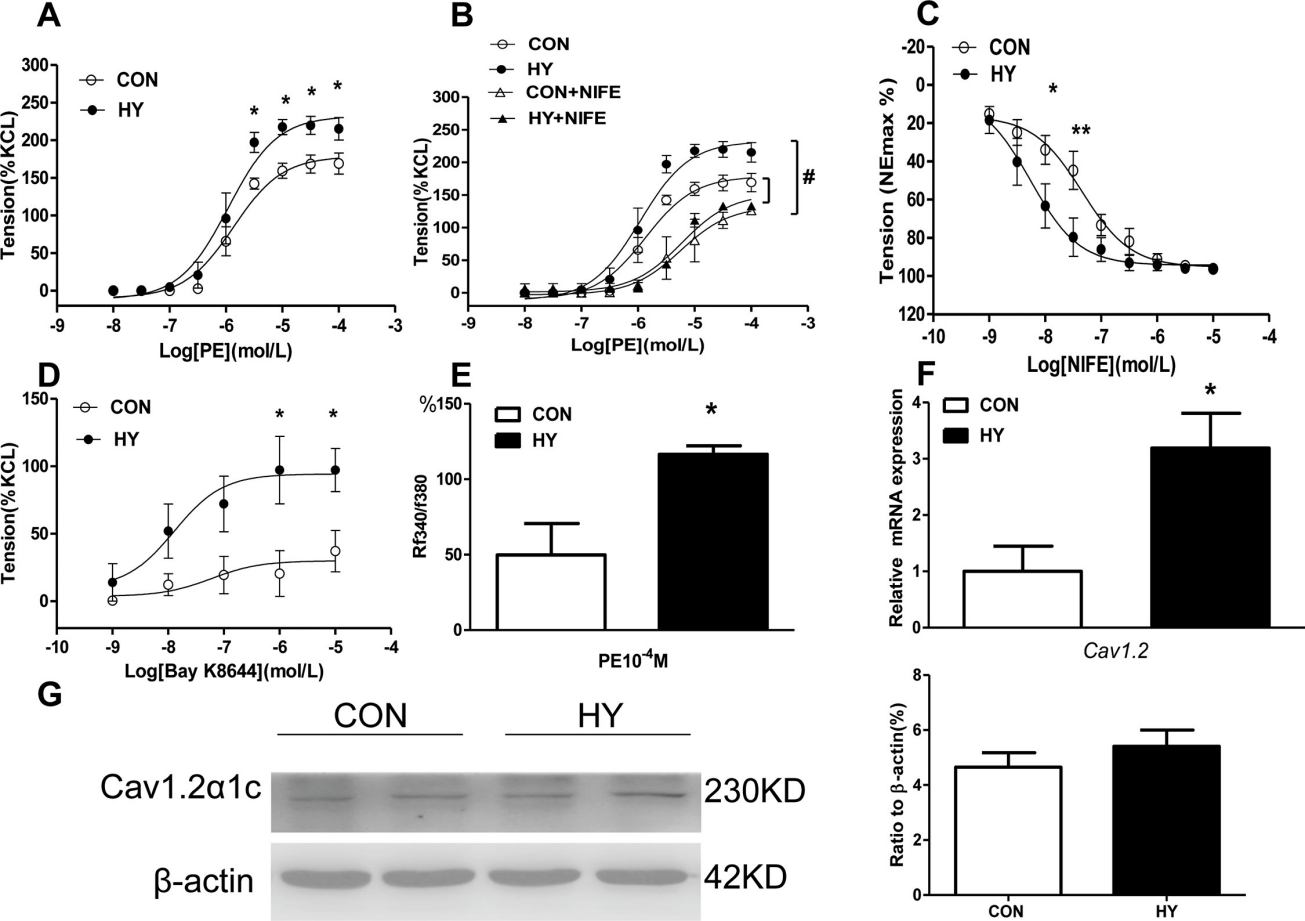
The effect of prenatal HY on contraction of mesenteric arteries in adult offspring. (A) Phenylephrine (PE)-induced vessel contractions in mesenteric arteries. Comparisons were made using Two-way ANOVA test. (n = 10, *P<0.05). (B) Suppression effect of nifedipine (NIFE) on PE-mediated contraction. Comparisons were made using Two-way ANOVA test. (n = 10, ^#^P<0.05). (C) Relaxation of NIFE on noradrenaline (NE) 10^−4^ M contracted response. Comparisons were made using Two-way ANOVA test. (n = 10, *P<0.05). (D) Vessel tension to Bay K8644 in mesenteric arteries. Comparisons were made using Two-way ANOVA test. (n = 10, *P<0.05). (D) Free intracellular calcium ([Ca^2+^]i) in response to phenylephrine (PE) 10^−4^ M in mesenteric arteries. Comparisons were made using two-tailed unpaired t-tests (n = 5, *P<0.05). (E) Relative mRNA expression of L-type calcium channel *(Cav1*.*2*) in mesenteric arteries. Comparisons were made using two-tailed unpaired t-tests (n = 10, *P<0.05). (F) Protein expression of Cav1.2α1c subunit in mesenteric arteries. Comparisons were made using two-tailed unpaired t-tests (n = 10).

We also characterized the contraction function of mesenteric arteries in both groups. PE-induced vessel tension in mesenteric arteries was greater in HY offspring (F_0.05(1,8)_ = 5.32<23.33, P<0.05, Fig 3A). And Fig 3B showed NIFE significantly depressed PE-induced contractions in both groups, while the decreased argument in HY group was greater than that in CON (F_0.05(3,8)_ = 4.07<28.94, P<0.05). Relaxation response to concentration-dependent NIFE in HY group was more sensitive than that in CON group (F_0.01(1,8)_ = 11.26<15.10, P<0.01<0.05, Fig 3C). Meanwhile, Bay K8644, an agonist for voltage dependent calcium channels, induced higher contraction response in HY group (F_0.05(1,4)_ = 7.71<20.81, P<0.05, Fig 3D). The change in [Ca^2+^]_i_ flux in offspring mesenteric arteries induced by PE was significantly greater in HY group than that in CON group (P<0.05, Fig 3E). The relative mRNA expression level of *Cav1*.*2* was increased significantly in HY group (Fig 3F). Cav1.2α1c protein analysis showed a trend of augmentation in mesenteric arteries of the HY, without significant statistical difference between both groups (Fig 3G).

### The effect of prenatal HY on RhoA/Rho kinase signaling pathway of mesenteric arteries in adult offspring

**Fig 4:**
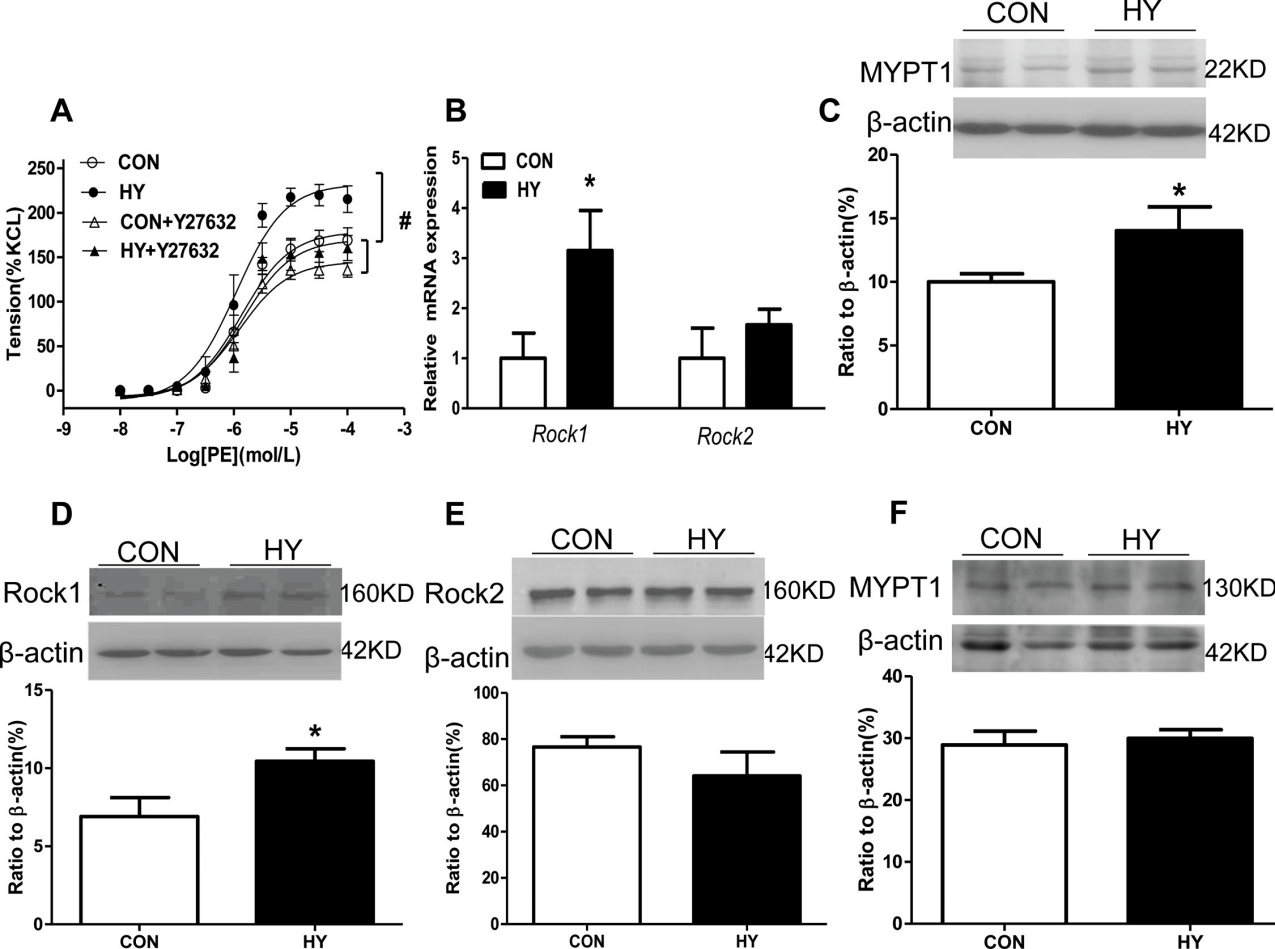
The effect of prenatal HY on RhoA/Rho kinase signaling pathway of mesenteric arteries in adult offspring. (A) PE-increased vasoconstriction in presence or absence of Y27632. Comparisons were made using Two-way ANOVA test (n = 10, ^#^P<0.05). (B) Relative mRNA expression of Rock isoforms in mesenteric arteries. Comparisons were made using two-tailed unpaired t-tests (n = 10, *P<0.05). Relative protein expressions of RhoA (C), Rock1 (D), Rock2 (E), and MYPT1 (F) in mesenteric arteries. Comparisons were made using two-tailed unpaired t-tests (n = 10, *P<0.05).

In order to better understand the altered function of mesenteric arteries by prenatal HY, the RhoA/Rho kinase signaling pathway was also tested. Rock antagonist Y27632 inhibited PE-induced contraction in mesenteric arterioles in both groups, with significant greater inhibition level in HY group (F_0.05(3,8)_ = 4.07<19.60, P<0.05, Fig 4A). In HY group, *Rock1* showed a no-significant up-ward tendency, and *Rock2* remained unchanged (Fig 4B). Protein analyses of RhoA was increased in HY group (Fig 4C) and Rock1 was increased significantly (Fig 4D), while Rock2 and MYPT-1 appeared unchanged (Fig 4E and 4F).

### The effect of prenatal HY on vascular structure in the SHR offspring

**Fig 5:**
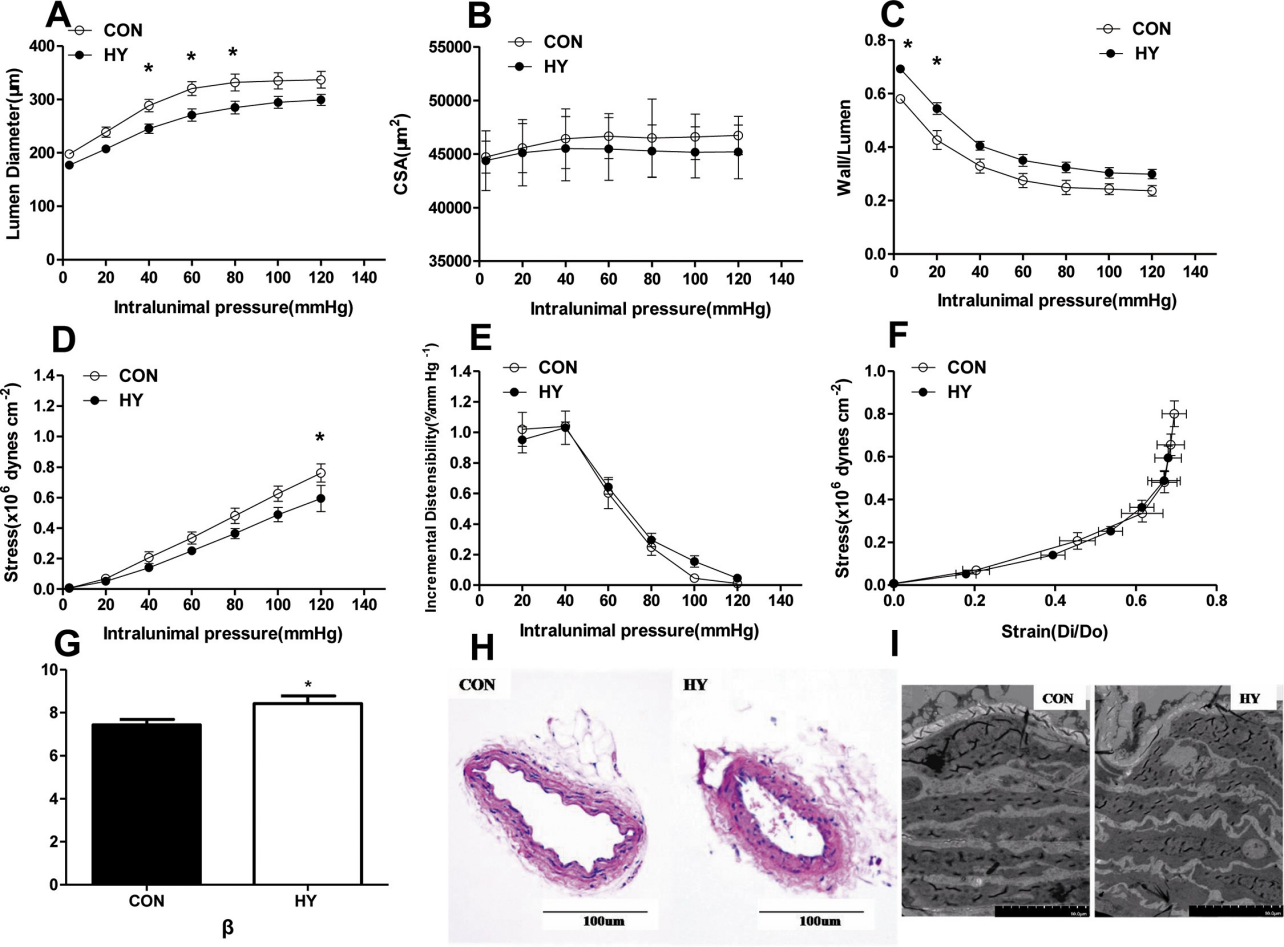
The effect of prenatal HY on vascular structure in the SHR offspring. Lumen diameter–intraluminal pressure (A) cross-sectional area (CSA)–intraluminal pressure (B) and wall/lumen ratio–intraluminal pressure (C) stress–intraluminal pressure (D) incremental distensibility–intraluminal pressure (E) and stress–strain (F) curves in mesenteric arteries from 12-week-old offspring. Comparisons were made using Two-way ANOVA test (n = 10, *P<0.05). (G) The parameter representing the degree of vascular stiffness. Comparisons were made using two-tailed unpaired t-tests (n = 10). (H) Hematoxylin and eosin stain sections in mesenteric arteries. Mesenteric vessels of HY groups show a thicker vascular media and the smooth muscle cells (SMC) were proliferative and arranged in disorder (Scale bars: 100 μm). (n = 5) (I) Vascular sections of transmission electron microscope. A thicker smooth muscle cell (SMC) layers and a significantly waving internal elastic lamina were observed in mesenteric vessels of HY groups (Scale bars: 10 μm). (n = 5).

Lumen diameter was significantly different between HY and CON offspring at 40-to-80mmHg pressure (F_0.05(1,6)_ = 5.99<42.53, P<0.05, Fig 5A), though the wall of CSA was similar in both groups (Fig 5B). Moreover, wall-to-lumen ratio was increased in HY as compared to CON (F_0.05(1,6)_ = 5.99<50.38, P<0.05, Fig 5C). And wall thickness of mesenteric arteries in HY showed no notable differences compared to CON (data not shown). Media stress was significantly smaller in HY than that in CON at 120mmHg pressure (F_0.05(1,6)_ = 5.99<14.90, P<0.05, Fig 5D), while incremental distensibility (Fig 5E) showed no differences between the two groups. There was an overlap in stress–strain curve (Fig 5F). HY group showed a larger value of β (CON: 7.44±0.35; HY: 8.42±0.25; P<0.05, Fig 5G).

Histological analysis for HE staining sections showed a thicker vascular media with smooth muscle cells arranged in disorder in HY group compared with CON group (Fig 5H). TEM analysis also demonstrated disorganized smooth muscle cell layers and matrix in the vascular tissue from HY group (Fig 5I).

## Discussion

Essential hypertension is the form of “hypertension” without identifiable cause as its definition, affecting more than 90% of hypertensive patients [26,27]. Usually appearance of hypertension is shown in patients in adult stages. It is well recognized that genetic and environmental interactions are basic hypothesis for development of essential hypertension. The major idea of the present study was to use a hypertension sensitive genetic model with special environmental treatments during critical early developmental period, to determine whether and how appearance of hypertension would move to younger ages. The results showed that the SHR offspring exposed to prenatal hypoxia exhibited greater risks in development of hypertension at adolescent age, as evidenced by enhanced resistance of small mesenteric arteries in early life periods. Importantly, the finding demonstrated that hypertension genetic model plus prenatal environmental insults such as hypoxia during pregnancy would make worse conditions and consequences for the vascular development, contributing to onset of essential hypertension in early life of the young offspring, proving that environmental factors during critical developmental periods play important roles in vascular dysfunction as well as hypertension in individuals with genetic defects.

It is well known that prenatal hypoxia could induce IUGR or low birth weight [28]. Epidemiology studies have demonstrated that IUGR or low birth weight can increase risks of cardiovascular impairment in later life [29,30]. Notably, body weight in SHR offspring has been connected with blood pressure levels [31]. In the present study, HY offspring showed a stable low body weight curve before 3-month-old with significant differences at 8 and 12 weeks compared to CON, demonstrating those young rats were unable to catch up body weight during juvenile period (roughly 1- to 3-month-old). Previous studies demonstrated that prenatal adverse factor-induced IUGR offspring had a catch-up growth after birth [29,30]. However, those offspring were not born from genetically hypertensive mothers. Present study demonstrated that SHR genetic factors might play roles in suppression of a catch-up growth following prenatal hypoxia-induced IUGR. Although cardiac or renal abnormalities were always complicated with malignant blood pressure in SHR offspring, in the present study, the weight of critical organs such as the heart and kidney were shown none of significant differences at 12-week-old between the two groups, as other species of rats in previous studies [32].

The SHR is a model of essential hypertension that is involved in an increase in total peripheral resistance with structural and functional modifications in mesenteric vessels [33–35]. After 13-week-old, systolic pressure in male SHR rats averages 180 mmHg or more due to genetic defects [33,34]. The present study was the first to investigate the effect of prenatal hypoxia on BP in young SHR offspring. Notably, BP at all measuring points appeared higher in the SHR young offspring compared to the control at same ages. For example, systolic pressure for SHR offspring in CON was under 150 and 170 mmHg at 6 and 8 weeks old. In comparison, the SHR offspring exposed to prenatal hypoxia showed higher BP (over 150 or 170 mmHg) at the same ages, while significantly statistical difference was confirmed at 12 weeks old. Thus, prenatal hypoxia contributed significantly to early onset of hypertension and BP aggravation in SHR young offspring. Telemetry is an excellent method to measure BP in free moving rats, which could significantly reduce influence by non-specific factors such as stress. It should be better if telemetry measurements were applied for the experiments. Since essential hypertension is recognized as a more common form in risk family of hypertension, the result provides new clues to explain development of hypertension in young kids, and suggests that special attention should be paid to avoid causes that may induce hypoxia during pregnancy for the mothers with essential hypertension. In addition, for those children born from mothers with essential hypertension and pregnant complications such as preeclampsia that may induce *in utero* hypoxia, special attention should be considered for cardiovascular protections.

Vascular tone and peripheral vascular resistance are major determinants of arterial pressure as well as for development of hypertension. In normal BP mothers, such as Sprague-Dawley rats, peripheral vascular dysfunction of offspring induced by prenatal negative stimulus was extensively studied [17–21]. Notably, accumulating evidences have suggested a relationship between prenatal hypoxia and increased cardiovascular risks in postnatal life in adults, which could be attributed to anomalous vascular tone [36]. Vascular vasodilatation plays important roles in the pathophysiology of hypertension. Previous studies showed a remarkably impaired vasodilatation in mesenteric arteries in normal BP rodents exposure to prenatal hypoxia [18,37]. In the present study, endothelium-dependent and independent relaxations were assessed respectively. Vascular relaxation in response to ACh was attenuated in the MA from HY group. It is well known that ACh acts on M-receptors on vascular endothelial cells, stimulating production of NO via activation of eNOS enzyme, which, in turn, binding and acting at sGC in smooth muscle cells to cause vasodilatation [38,39]. The present study found that, following application of L-Name, an eNOS blocker, PE-mediated vascular responses were less affected in HY offspring than that in CON, indicating eNOS-mediated endogenous NO production might be damaged by hypoxia in the MA of SHR young offspring. Furthermore, molecular analysis showed a decreased mRNA expression of eNOS in the MA of HY offspring. Plasma NO levels were reduced while ET-1 was increased. Together, those data suggested that vascular endothelium may be damaged by prenatal hypoxia in SHR young offspring, evidenced with impaired key enzyme activities in endogenous NO system as well as ACh-mediated functional changes. Sodium nitroprusside (SNP), a classic exogenous NO donor, is frequently employed as a nitrovasodilator in vascular relaxation. Subsequent experiments in the present study used SNP to test vasodilatation independent of the endothelium. The results showed that vasodilatation was less sensitive to SNP in the MA of HY offspring, suggesting the decreased sensitivity to exogenous NO in VSMC by hypoxia. Together, these results provided evidence that prenatal hypoxia disturbed the pathway of NO producing and acting signaling pathways in both endothelial and smooth muscle cells in the MA of SHR young offspring, contributing to the impaired vasodilatation and enhanced vasoconstriction, resulting in a greater increase of BP.

Besides abnormal vasodilatation, dysfunction of vasodilatation also plays important roles in the pathophysiology of hypertension. The present study found PE-mediated vascular tension of MA was significantly higher in HY offspring than that of the control, indicating functional changes in resistance arteries following prenatal hypoxia may contribute to the increased BP in the adolescent offspring. Previous studies showed that hypoxia in pregnancy in normal BP mothers could affect vascular functions and structures in the adult offspring. The new finding in micro-vessels in the present study emphasized that genetic influence could make fetal vascular systems more vulnerable to environmental insults, leading to more damage during sensitive periods for vascular development. PE is a vascular agonist, binding to G-protein coupled receptors and inducing contraction by increasing both cytosolic Ca^2+^ concentrations and Ca^2+^ sensitivity of the contractile apparatus. In the Ca^2+^ concentration-dependent signaling cascades, Ca^2+^ influxes through voltage dependent calcium channels, binds to calmodulin, causing contraction in smooth muscle cells [40–42]. Our previous work have observed the altered functions of Ca^2+^ channels in various adult vessels after exposure to hypoxia [28] or high sucrose [20] during gestation. In the present study, PE induced much more [Ca^2+^]_i_ in vascular rings from HY offspring, indicating that the Ca^2+^ channels in resistance vessels from SHR offspring were affected by prenatal hypoxia. Since L-type Ca^2+^ channels (Cav1.2) are dominant in the MA [40] and the enhanced Cav1.2 functions were well documented in SHR [43,44], we thus applied the specific agonist and inhibitor to investigate possible functional changes in the MA. Bay K8644 caused greater vasoconstrictions, while nifedipine induced larger suppression in PE-mediated contractions in HY offspring compared with CON. The mRNA expression of Cav1.2α1c subtype in the MA of HY offspring was significantly increased, while the protein expression appeared higher than that of CON, without statistical difference. These results demonstrated that, in the MA of young SHR offspring exposed to prenatal hypoxia, voltage-dependent calcium channels underwent molecular and functional changes, causing a greater intracellular flux of [Ca^2+^]_i_, and inducing higher vascular contractile responses.

The Ca^2+^-sensitizing effect of vasoconstrictors is ascribed to RhoA activation and the subsequent stimulation of its target Rho kinase, which phosphorylates myosin phosphatase-targeting subunit 1 (MYPT-1), the regulatory subunit of MLCP, and thereby inhibits MLCP activity, resulting in vascular contraction [45,46]. The activity of RhoA/Rho kinase signaling pathway was enhanced in microvascular tissue following maternal hyperglycemias, and the inhibition could attenuate acute hypoxic fetoplacental vasoconstriction in rats. The present study used the Rho kinase inhibitor to test possible alterations in RhoA/Rho kinase signaling. Incubation with Y27632 significantly reduced α_1_ adrenoreceptor-mediated contractions in the MA, with an augmented decrease in HY group. There are two isoforms for Rho kinase, Rock1 and Rock2, both expressed in the MA. Although relatively few studies have outlined specific roles of each isoforms, Rock1 was considered to interact with MYPT-1 twice more than that of Rock2 [47]. Rock2 was suggested to be predominant in VSMC contractility and critical in the hypoxia-induced pulmonary hypertension. In the present study, both mRNA and protein expression of Rock1 were significantly increased in HY group, while Rock2 remained unchanged. In addition, we measured the protein expression of the upstream molecule RhoA and downstream main substrate MYPT1. Interestingly, RhoA was increased, and MYPT-1 was unchanged. Those new data demonstrated that RhoA and Rock1, in RhoA/Rho kinase signaling pathway, were altered by prenatal hypoxia in the SHR offspring, which might contribute to the different vascular responses in the MA by Y27632-mediated Rho kinase inhibition. Further investigations should be continued to identify precise functions of Rock isoforms involved. Despite that, the finding offered important information on underlying mechanisms (impaired RhoA/Rho-Cav1.2 signaling pathway) for the increased PE-mediated vasoconstrictions by prenatal hypoxia in the young SHR offspring.

It is well accepted that hypertension is usually associated with structural changes in the resistance vasculature [48]. The main feature of altered mechanical properties of small arteries in hypertensive vascular-remodeling is an increased wall to-lumen ratio, and this parameter has a prognostic value for cardiovascular events [49]. Maternal hypoxia was documented to induce increased micro-vascular stiffness in CD1 mice [19] and early morphological changes of atherosclerosis in adult SD offspring [50]. In the present study, the MA from HY offspring showed eutrophic inward remodeling compared with CON group, with unchanged cross-sectional area and increased wall-to-lumen ratio. The vessels from HY offspring also showed increased wall thickness. Notably, prenatal hypoxia showed an altered vascular stiffness as the β values were increased in the present study. Vascular smooth muscle cell proliferation is an important cellular basis of vascular remodeling and the stiffness is greatly influenced by the extracellular matrix [51]. Analysis of ultra structures of vessels revealed that HY group showed aggravated disorder in vascular smooth muscle cells and excessive and aberrant matrix deposition, providing structural clues for the altered mechanical properties observed. The findings of subtle morphological changes or vascular tissue remodeling in the MA by prenatal hypoxia provided interesting evidence that resistance vessels in SHR subjects were more vulnerable to environmental insults during pregnancy, enhancing vascular damage associated with genetic defects, which may increase incidence of hypertension after birth compared with the control SHR offspring.

This study was the first to show that prenatal adverse environments such as hypoxia could move inherited hypertension from usual adult stages to younger age. Possible underlying mechanisms may be related to the altered Ca^2+^-mediated vascular constrictions and endothelial vasodilatation in the young SHR offspring exposed to prenatal hypoxia. The results provided new evidence that interactions between genetic factors and environmental insults during fetal periods could increase cardiovascular risks and induce hypertension in young kids with genetic defects. The finding suggests if attention could be paid to reduce or remove environmental adverse influence, inherited hypertension would be preventable, at least in the young populations. Further investigations in that direction would be important for further understanding underlying mechanisms of essential hypertension in developmental origins, as well as for finding targets for early prevention or treatments.
